# Assessment of superior vena cava diameter and collapsibility index in liver transplantation: a prospective observational study

**DOI:** 10.1016/j.bjane.2024.844563

**Published:** 2024-10-09

**Authors:** Maged Y. Argalious, Sven Halvorson, John Seif, Sandeep Khanna, Mi Wang, Jacek B. Cywinski

**Affiliations:** a Cleveland Clinic, Integrated Hospital Care Institute, Department of Anesthesiology, Multispecialty Anesthesiology, Cleveland, USA; b University of Oregon, Prevention Science Institute, Oregon, USA

**Keywords:** Central venous pressure, Echocardiography, Liver transplantation, Vena cava, Superior

## Abstract

**Background:**

Superior Vena Cava (SVC) diameter and collapsibility index, dynamic measures of fluid responsiveness, have been successfully utilized as echocardiographic indices for fluid responsiveness in ventilated septic patients. Whether these measurements are correlated with Central Venous Pressure (CVP) measurements in liver transplant patients is unknown. We sought to assess the correlation of maximum and minimum SVC diameter and SVC collapsibility index measurements obtained intraoperatively by Transesophageal Echocardiography (TEE) with those of simultaneously recorded CVP measurements obtained through a right atrial port of a pulmonary artery catheter. The secondary aim of the study was to assess the correlation between SVC measurements and simultaneously obtained thermodilution cardiac index measurements.

**Methods:**

Single center prospective observational trial of patients with end stage liver disease undergoing liver transplantation in an academic tertiary care center.

**Results:**

The minimum SVC exhibited a mild significant correlation with CVP as did the maximum SVC. The correlation between the SVC collapsibility index and CVP was not significantly different from zero. In our secondary analysis, the correlation between minimum SVC diameter and cardiac index was determined to be weak but non-zero as was the correlation between the maximum SVC diameter and cardiac index. The correlation between SVC collapsibility index and cardiac index was not different from zero.

**Conclusion:**

While statistically significant, the weak clinical correlation of intraoperative SVC measurements obtained by TEE make them unsuitable as a replacement for central venous pressure or thermodilution cardiac index measurements in liver transplant recipients.

## Introduction

Central Venous Pressure (CVP) is routinely measured during liver transplant surgery; it can be accomplished through a central venous catheter in the internal jugular vein or through a right atrial port of a Pulmonary Artery Catheter (PAC). Central venous pressure measurements at the Superior Vena Cava-Right Atrial (SVC-RA) junction are a function of circulating blood volume, right ventricle function, compliance, and intrathoracic pressure.^1^^,^^2^ In spontaneously breathing patients, transthoracic echocardiographic measurements of the Inferior Vena Cava (IVC) diameter and its change with inspiration (sniff) is routinely used to estimate right atrial pressure. This echocardiographic measurement allows noninvasive estimation of pulmonary artery systolic pressure based on the modified Bernoulli equation (PASP = RVSP = 4v^2^ + RA pressure).^3^ Due to its intraabdominal location, the IVC is less ideally suited for estimation of right atrial pressure during mechanical ventilation; especially because positive pressure ventilation causes a dilation of IVC diameter.^4^

Given the entirely intrathoracic location of the Superior Vena Cava (SVC), its diameter (normal diameter: 1.2–2.2 cm) and collapsibility with positive pressure ventilation, it is a potentially attractive method of noninvasively estimating CVP.^5^ The magnitude of SVC collapsibility is a function of the transmural pressure (intrathoracic pressure minus SVC pressure).

Superior vena cava diameter and collapsibility index, dynamic measurements of fluid responsiveness have been successfully utilized as echocardiographic indices for perioperative fluid responsiveness in open major vascular surgery patients,^6^ general surgery patients,^7^ and in critically ill ventilated septic patients.^8^ Whether SVC diameter and SVC collapsibility are correlated with CVP measurements in liver transplant patients is unknown. The primary aim of this study was to assess the correlation of maximum and minimum SVC diameter and SVC collapsibility index measurements obtained intraoperatively by Transesophageal Echocardiography (TEE) with those of simultaneously recorded CVP measurements obtained through a centrally inserted venous catheter or a right atrial port of a PAC. The secondary aim of the study was to assess the correlation between SVC measurements and simultaneously obtained thermodilution cardiac output and cardiac index measurements.

The principle of thermodilution cardiac output relies on a decrease in blood temperature in response to injecting a known volume of room temperature (cold) fluid into the right atrium through the central venous pressure port of the pulmonary artery catheter. This change is then measured by a thermistor in the pulmonary artery. The decrease in temperature is inversely proportional to the dilution of the injectate, or cardiac output.^9^ Since cardiac output is a product of stroke volume and heart rate, changes in SVC diameter and collapsibility may correlate with changes in stroke volume (another indicator for fluid responsiveness) and therefore cardiac output and index measurements.

Hypothesis: SVC diameter and SVC collapsibility index correlate with CVP measurement in recipients undergoing liver transplantation surgery.

## Methods

Ethical approval for this study (IRB#:16-307) was provided by the Institutional Review Board at Cleveland Clinic, Cleveland, OH (Executive director: Bridget Howard, Esq, CIP) on March 7^th^, 2016. Our study was registered at ClinicalTrials.gov (NCT02818218; principal investigator M.A.; date of registration June 29, 2016) before the enrollment of the first patient.

**Figure 1:**
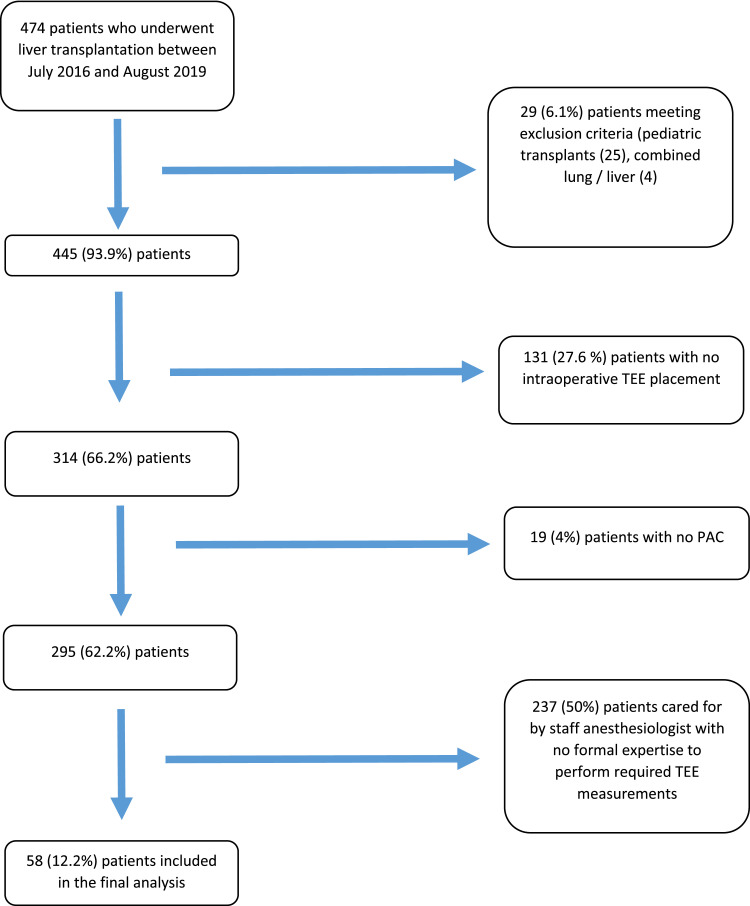
Flow diagram.

This was a single group prospective observational trial, and we included patients between 18 and 80 years of age undergoing living donor related and cadaveric orthotopic liver transplantation (recipient) surgery at the Cleveland Clinic. We excluded patients who had established contraindications to intraoperative TEE placement, including those with esophageal strictures and active gastrointestinal (variceal) bleeding.^10^ We excluded patients for whom PAC insertion was not anticipated during the surgery. Since intraoperative TEE measurements require advanced expertise in perioperative TEE, we excluded patients when the anesthesiology staff could not perform these measurements. We also excluded patients with combined lung liver transplantation and combined heart liver transplantation since those surgeries start with the lung and heart portion before the liver transplantation portion, thereby delaying the timing for measurements. In contrast, we did include combined liver kidney transplantation cases since the liver transplantation portion occurs first (Fig. 1).

Induction of anesthesia was achieved with fentanyl 2–4 mcg.kg^-1^ and either propofol 1–2 mg.kg^-1^ or etomidate 0.2–0.3 mg.kg^-1^. Neuromuscular blockade to facilitate endotracheal intubation was achieved with succinylcholine 1 mg.kg^-1^ or rocuronium 0.6–0.9 mg.kg^-1^. Maintenance of anesthesia was achieved with isoflurane in oxygen/air mixture and additional doses of intravenous (IV) fentanyl were administered throughout the case. Maintenance of neuromuscular blockade was achieved with intermittent doses of rocuronium to maintain a moderate to deep level of neuromuscular blockade throughout the case.

Patients were mechanically ventilated using pressure control ventilation with a tidal volume of 6–8 mL.kg^-1^ of ideal body weight with a respiratory rate of 10–12 min and a positive end expiratory pressure of 5–8 cm H_2_O, an inspiratory to expiratory ratio of 1:2 and a fraction of inspired oxygen of 0.5. Peak inspiratory pressures were maintained at less than 35–40 cm H_2_O.

Fluid management was guided by invasive hemodynamic data, including measurements of fluid responsiveness including arterial line derived pulse pressure variations and TEE guided stroke volume variations. Red blood cell transfusion was guided by serial blood gas analysis to maintain hematocrit levels above 24 g.dL^-1^ and thromboelastography was used to guide blood product transfusion. Crystalloids (Plasmalyte and normal saline) were used sparingly and albumin 5% was used as the colloid of choice.

Measurements of SVC diameter and SVC collapsibility index were recorded by anesthesiologists with expertise and qualifications in basic or advanced TEE.

**Figure 2:**
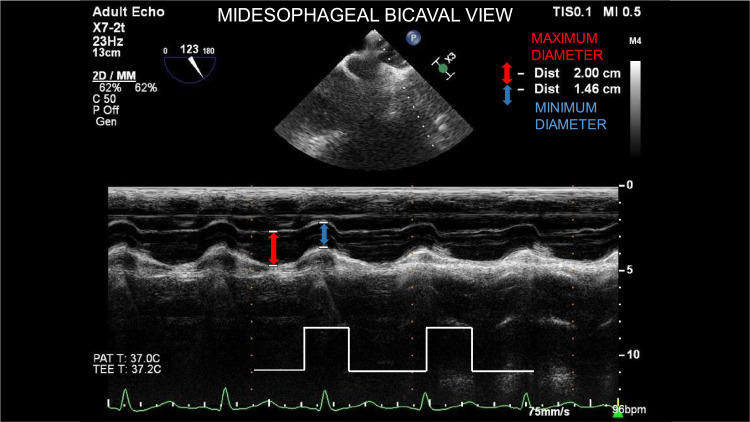
M mode assessment of maximum and minimum Superior Vena Cava diameter utilizing Trans-Esophageal bicaval view at 123 degrees. Positive pressure ventilation and the subsequent increase in intrathoracic pressure causes a reduction in SVC diameter.

Specifically, we utilized M mode echocardiography to measure the diameter of the superior vena cava using Philips Affinity 70°C TEE machine. The TEE probe was placed at the level of the mid-esophagus and the omni-plane was adjusted to around 110 degrees to obtain the bicaval view (Fig. 2). This technique is analogous to that recommended when measuring IVC diameter and collapsibility.^3^^,^^5^ The maximum and minimal diameter of the SVC were measured over one respiratory cycle and recorded in real time. SVC collapsibility index, which is a measure of the inspiratory decrease in SVC diameter was calculated based on the following formula $\text{SVC}\;\text{Collapsibility}\;\text{Index}=100\cdot \frac{SVC_{max}-SVC_{min}}{SVC_{max}}$

Simultaneously, central venous pressure measurements were recorded from the right atrial port of the pulmonary artery catheter. In the supine position, patients’ pressure transducers were zeroed to atmospheric pressure and located at the mid-thoracic position, as previously described.^11^ Simultaneous manual intermittent thermodilution cardiac output and index measurements were also obtained and recorded during the same time periods using a 7.5 F Bioptimal pulmonary artery catheter and a 10 mL Edwards Lifesciences cardiac output set and closed injectate delivery system for room temperature.

The hemodynamic measurements including minimum and maximum SVC diameter, central venous pressure, and the cardiac output and index were taken intraoperatively during the following five time periods: post-induction of anesthesia defined as the time period following endotracheal intubation and invasive line placement until surgical incision, pre-anhepatic phase: beginning with surgical incision and ending with surgical clamping of the portal vein, hepatic artery, and inferior vena cava; anhepatic phase: begins when the hepatic venous inflow is clamped and ends when the graft (new liver) is reperfused; post-anhepatic phase: begins when the liver is reperfused and flow resumes in the inferior vena cava and portal vein, and the last phase which begins with surgical closure of the deep fascial layer of the anterior abdominal wall and ends with completion of surgery.

Hemodynamic measurements were recorded on a paper report form for each individual patient and were later filed into a Redcap database. Patient demographics, medical history, laboratory values, and intraoperative factors were collected from our Electronic Medical Records (EMR) as well as from our Perioperative Health Documentation Database System (PHDS).

**Figure 3:**
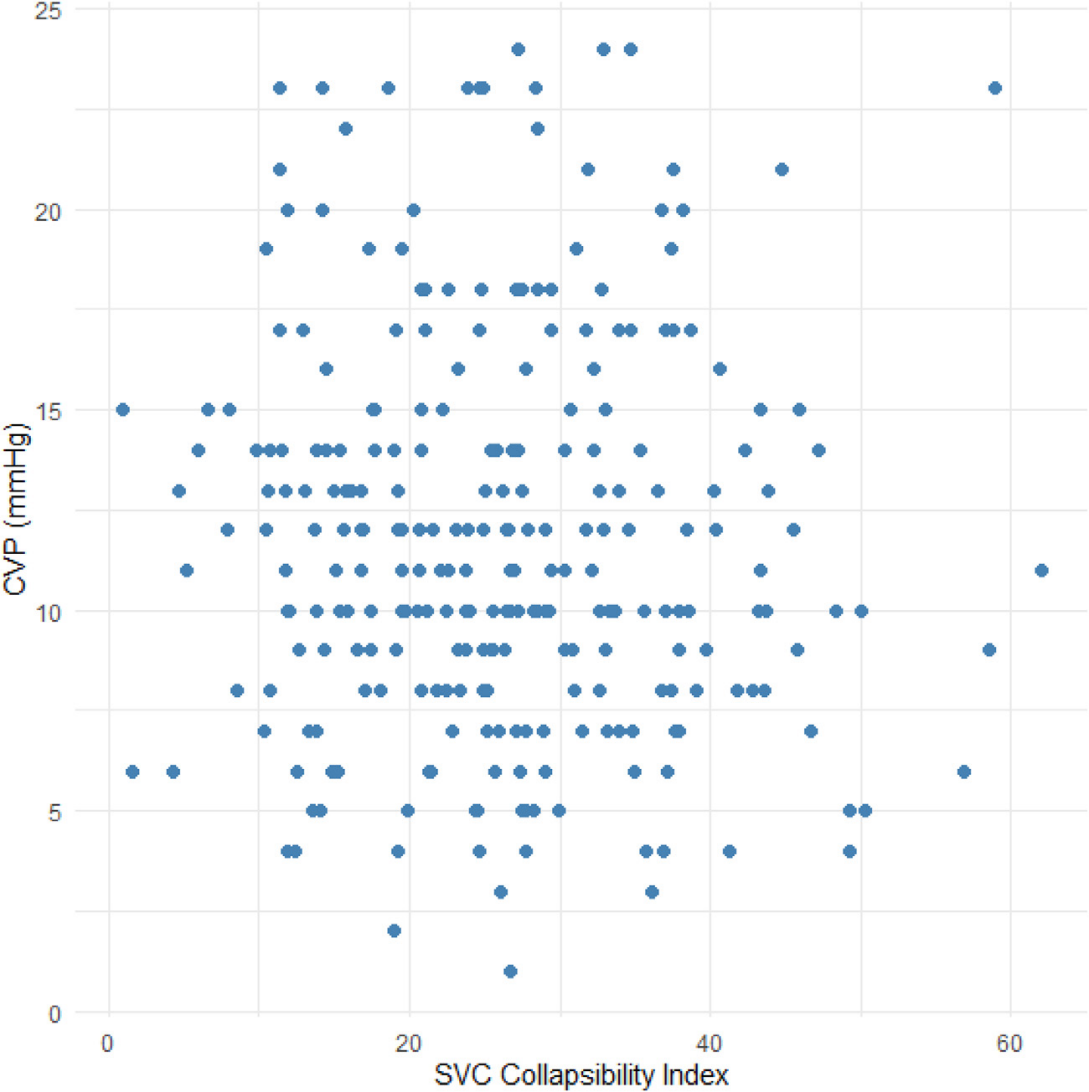
**CVP is plotted against the SVC collapsibility index**. The repeated measures correlation was not different from zero (*r_rm_* (98.3% CI): 0.005 (-0.161, 0.170), *p* = 0.944).

Our primary aim was to assess the correlation between the three SVC measurements (collapsibility index, minimum and maximum diameters) and CVP. (Fig. 3). We computed the repeated measures correlation^12^^,^^13^ (rmcorr or $r_{rm}$) between each of the SVC measurement and CVP. This method was chosen over Pearson's correlation coefficient to account for possible intra-subject correlation and lack of independence. Repeated measures correlation is similar to ANCOVA but fits the patient as a factor. These correlations were tested using α = 0.05/3 = 0.017 significance criteria to correct for multiple comparisons.

To guard against bias due to missing data, a sensitivity analysis was conducted on the subset of the population with complete SVC and CVP measurements across all five operative periods.

In an additional sensitivity analysis, we used bootstrap resampling^14^ to assess the robustness of the repeated measures correlation analysis in adjusting for the within-subject correlation. Specifically, for each of 10,000 bootstrap datasets we randomly sampled patient IDs, with replacement, and computed Pearson's correlation between the SVC measurements and CVP across the 5 time periods. We then estimated a 98.3% confidence interval for the correlation of interest from the 1.7^th^ and 98.3^rd^ percentiles of the bootstrap resamples.

For our secondary analysis, we estimated $r_{rm}$ between cardiac index and the three SVC measurements.

In addition to our primary and secondary hypotheses, we also examined potential differences in the SVC-CVP relationship by surgical period as an exploratory aim. The analysis was conducted by fitting linear mixed effects models using unstructured correlation. These models used the SVC measurements, the surgical period, and their interaction as predictors and CVP as the response.

### Sample size, precision, and power

Initially, a sample size of 100 patients was planned to obtain sufficiently narrow widths of the correlation between the three SVC measurements (minimum, maximum, and collapsibility index) and CVP. With zero intra-subject correlation and complete data, the width of a 98.3% Pearson's confidence interval would be 0.21 for a correlation of 0.5, and 0.36 for an intra-subject correlation of 1. Due to much slower than expected enrollment, the decision was made at n = 25 to continue to an n of 50, since this would still give adequate precision and power. With the final sample size of 58 patients, the correlation confidence interval was expected to have a width of 0.27 with no intra-subject correlation, and slightly wider with modest intra-subject correlation.

A post-hoc power analysis showed that with the 268 measurements from 58 participants we had 98% power to detect a repeated measures correlation of 0.3 or greater (with correlations less than 0.30 not deemed clinically important). This was computed from the degrees of freedom for the repeated measures correlation (9) as $n\left(k-1\right)-1=58\left(\frac{268}{58}-1\right)-1=\;$209. This was then used to compute the power for a Pearson's correlation test using a significance criterion of α = 0.017.

## Results

### Participants

Between 2016 and 2019, a total of 58 patients scheduled for liver transplantations were recruited for the study and their hemodynamics were recorded.

Patient demographics, relevant medical history, and intraoperative data are summarized in Table 1. Across all periods, the population had a mean ± S.D. CVP of 11.7 ± 4.9 mmHg, an SVC collapsibility index of 26.0 ± 11.0, a minimum SVC diameter of 12.8 ± 3.1 mm, and a maximum SVC diameter of 17.3 ± 3.2 mm. These hemodynamic measurements are summarized in Table 2.

**Table 1:** Cohort description.

Variable	Measurement
Number	58
**Demographics**	
Age (years)	60 [55, 67]
Female (%)	17 (29)
White (%)	48 (83)
Body Mass Index	29 ± 6
Current smoker (%)	26 (46)
American Society of Anesthesiologists physical status (%)	
III	3 (5.2)
IV	53 (91)
V	2 (3.4)
**Etiology of liver disease (%)**^a^	
Alcoholic cirrhosis	18 (31)
Primary biliary cirrhosis	1 (1.7)
Hepatocellular carcinoma	17 (29)
Primary sclerosing cholangitis	7 (12)
Non-alcoholic steatohepatosis (NASH)	39 (67)
Viral cirrhosis	16 (28)
**Comorbidities (%)**^b^	
Acute cerebrovascular disease	7 (12)
Deficiency anemia	34 (59)
Coronary artery disease	8 (14)
Congestive heart failure	2 (3.4)
Chronic obstructive pulmonary disease	13 (22)
Diabetes Mellitus	19 (33)
Hypertension	34 (59)
Hypothyroidism	9 (16)
Neurological disorders	23 (40)
Paralysis	0 (0)
Peripheral vascular disease	22 (38)
Pulmonary circulation disease	10 (17)
Renal failure	23 (40)
**Preoperative labs**	
Albumin (mg.dL^-1^)	3.1 ± 0.6
Creatinine (mg.dL^-1^)	1.10 [0.80, 1.60]
Hematocrit (%)	30 [26, 37]
Model for End Stage Liver Disease-Na Score	21 [15, 27]
**Intraoperative factors**	
Liver-kidney combined transplant (%)	4 (6.9)
Surgical Duration (hrs.)	10.3 ± 2.0
Peak Inspired Pressure TWA (cm H_2_0)	22.3 [19.2, 26.1]
Positive End Expiratory Pressure TWA (cm H_2_0)	5.00 [4.50, 5.00]
Crystalloid fluids (L)	3.5 [2.9, 4.5]
Colloid fluids (L)	3.6 [3.1, 5.0]
Blood Loss (mL)	4000 [2500, 9000]
Red blood cells (units)	0.0 [0.0, 2.8]
Fresh frozen plasma (units)	0 [0, 0]
Platelets (units)	0.0 [0.0, 1.8]
Cryoprecipitate (units)	0 [0, 0]

Summary statistics are given as mean ± S.D., median [Q1, Q3], or count (%).

a Etiology of liver disease was determined by preoperative International Classification of Disease codes and were not mutually exclusive.

b Comorbidities reflect patient medical history for one year prior to surgery. TWA, Time Weighted Average.

**Table 2:** Hemodynamics summary.

Measurement	N^a^	Mean	S.D.	Median [Q1, Q3]	Missing %
SVC Collapsibility Index	269	26.0	11.0	25.5 [17.6, 33.0]	7.2
Minimum SVC (mm)	269	12.8	3.1	12.3 [10.9, 14.3]	7.2
Maximum SVC (mm)	269	17.3	3.2	17.1 [15.3, 19.1]	7.2
Central Venous Pressure (mmHg)	275	11.7	4.9	11.0 [8.0, 14.0]	5.2
Cardiac Output (L.min^-1^)	273	8.9	3.1	8.9 [6.4, 10.8]	5.9
Cardiac Index	269	4.4	1.5	4.3 [3.3, 5.4]	7.2

Hemodynamics are summarized as mean, standard deviation, median [Q1, Q3], and the percent of missing observations.

a Number of measurements taken from a total of 58 patients. SVC, Superior Vena Cava.

### Statistical analyses

Our primary analysis was to test the repeated measures correlation between CVP and the three SVC measurements. The correlation between SVC collapsibility index and CVP was not significantly different from zero ($r_{rm}$ (98.3% CI): 0.005 (-0.161, 0.170), *p* = 0.944).^15^ The minimum SVC exhibited a mild correlation with CVP ($r_{rm}$ (98.3% CI): 0.27 (0.11, 0.42), *p* < 0.001) as did the maximum SVC ($r_{rm}$ (98.3% CI): 0.34 (0.19, 0.48), *p* < 0.001).^16^ These results are summarized in Table 3.

**Table 3:** Primary analysis.

Measurement 1	Measurement 2	r_rm_ (98.3% CI)	*p*	n
Central Venous Pressure (mmHg)	SVC Collapsibility Index	0.005 (−0.161, 0.170)	0.944	268
Central Venous Pressure (mmHg)	Minimum SVC (mm)	0.27 (0.11, 0.42)	<0.001	
Central Venous Pressure (mmHg)	Maximum SVC (mm)	0.34 (0.19, 0.48)	<0.001	

Repeated measures correlations were computed between CVP and each of the three SVC measurements. A significance criterion of α = 0.017 was used.

SVC, Superior Vena Cava.

There was a total of 22 (8%) observations with one or more missing measurements across 16 (28%) patients. As a result, a sensitivity analysis was conducted where the repeated measures correlations were re-computed with only the 42 patients who had complete CVP and SVC measurements across all five surgical periods. This analysis as well as our bootstrapped confidence intervals were consistent with the primary analysis and are listed in Supplemental Tables 1 and 2. Supplemental Table 3 includes a STROBE checklist.^17^

Our secondary analysis, computing $r_{rm}$ between the three SVC measurements and cardiac index produced similar results as our primary analysis. The correlation between SVC collapsibility index and cardiac index was not different from zero ($r_{rm}$ (98.3% CI): 0.07 (-0.10, 0.24), *p* = 0.298). The correlation between minimum SVC diameter and cardiac index was determined to be weak but non-zero ($r_{rm}$ (98.3% CI): 0.22 (0.05, 0.37), *p* = 0.002) as was the correlation between the maximum SVC diameter and cardiac index ($r_{rm}$ (98.3% CI): 0.33 (0.17, 0.47), *p* < 0.001). These analyses are given in Table 4.

**Table 4:** Secondary analysis.

Measurement 1	Measurement 2	r_rm_ (98.3% CI)	*p*	n
Cardiac Index	SVC Collapsibility Index	0.07 (−0.10, 0.24)	0.298	262
Cardiac Index	Minimum SVC (mm)	0.22 (0.05, 0.37)	0.002	
Cardiac Index	Maximum SVC (mm)	0.33 (0.17, 0.47)	<0.001	

Repeated measures correlations were computed between cardiac index and each of the three SVC measurements. A significance criterion of was used.

SVC, Superior Vena Cava.

Our exploratory analysis looked at interactions between SVC predictors and the five surgical periods. These models did not detect any significant variations in the interaction between SVC measurements and CVP changes by surgical period. The results of these analyses are given in Table 5.

**Table 5:** Exploratory analysis.

Primary Predictor	Period	Slope (98.75% C.I.)	*p*-value	Interaction *p*-value
SVC Collapsibility Index	Post-induction	−0.01 (−0.11, 0.09)	0.84	0.08
	Pre-anhepatic	−0.08 (−0.22, 0.07)	0.19	
	Anhepatic	−0.08 (−0.23, 0.08)	0.22	
	Post-anhepatic	0.03 (−0.12, 0.19)	0.59	
	Post-closure	0.06 (−0.07, 0.20)	0.25	
Minimum SVC (mm)	Post-induction	0.21 (−0.14, 0.57)	0.13	0.12
	Pre-anhepatic	0.33 (−0.11, 0.77)	0.06	
	Anhepatic	0.4 (−0.2, 1.0)	0.10	
	Post-anhepatic	0.1 (−0.4, 0.6)	0.69	
	Post-closure	−0.1 (−0.6, 0.4)	0.55	
Maximum SVC (mm)	Post-induction	0.22 (−0.09, 0.54)	0.07	0.56
	Pre-anhepatic	0.24 (−0.19, 0.67)	0.16	
	Anhepatic	0.2 (−0.3, 0.8)	0.34	
	Post-anhepatic	0.26 (−0.23, 0.74)	0.18	
	Post-closure	0.11 (−0.38, 0.60)	0.57	

Mixed effects models were fitted with CVP as the response and SVC measurements, the surgical period, and their interactions as predictors. The slopes given are the change in CVP corresponding to a 1unit change in the corresponding SVC measurement.

SVC, Superior Vena Cava; CVP, Central Venous Pressure.

## Discussion

The results of this study show a statistically significant weak positive correlation between both minimum SVC measurements: 0.34 (0.19, 0.48), maximum SVC measurements: 0.27 (0.11, 0.42) and CVP. To put it in perspective, a strong positive repeated measure correlation is between 0.75 and 1 and a value close to 0 indicates no correlation. A positive repeated measure correlation indicates that SVC measurements are directly related to CVP measurements, meaning that as the SVC diameter increases, the CVP also increases.

These weak positive correlations, despite achieving statistical significance, are clinically not convincing to reliably estimate central venous pressure measurements in liver transplant surgery. Consequently, TEE-based measurements of maximum and minimum SVC diameter cannot replace right atrial pressure (central venous pressure) measurements for calculation of pulmonary artery systolic pressure during liver transplant surgery.

Our results are similar to those by Cowie et al.,^5^ who studied the correlation between SVC measurements and CVP in cardiac surgery patients and identified that noninvasive estimation of CVP in cardiac surgery patients cannot be achieved with TEE measurements of SVC diameters.

In contrast, a recent study correlating internal jugular vein collapsibility with CVP in patients with liver cirrhosis showed good correlation.^18^ Their medical study population excluded patients with mechanical ventilation and those with acute bleeding, indicating that point of care ultrasound central vein collapsibility measurements may better correlate with invasively measured central venous pressure measurements in the absence of hemodynamic and fluid shifts.

If our study results had shown a strong positive correlation between SVC diameters and CVP, it could have resulted in less reliance on central invasive catheter use in a select population of liver transplant recipients, especially those with normal pulmonary artery pressures and normal right ventricle function. Large bore peripheral venous catheters that allow rapid administration of fluids, blood products, and vasopressors would substitute central venous catheter use with TEE monitoring of SVC diameters as well as for calculation of Pulmonary Artery Systolic Pressure (PASP). In addition, a previous study showed that peripheral venous pressure correlates with CVP even under adverse hemodynamic conditions in patients undergoing liver transplantation.^19^

In contrast, CVP measurements, although unhelpful as a measurement of fluid responsiveness, remain an important hemodynamic component during liver transplant surgery,^20^ since a rise in CVP especially during the postanhepatic phase can alert the perioperative team to elevation of pulmonary artery pressure and/or a decline in right ventricle function.^21^ This in turn triggers several therapeutic interventions aimed at improving right ventricle contractility and at reducing an increasing afterload on the newly transplanted liver, which can otherwise result in liver congestion, increased bleeding and coagulopathy, and delayed function of transplanted graft.^22^

Our study results also showed a nonsignificant correlation between SVC collapsibility index and CVP measurements. This finding was not surprising since minimum and maximum SVC measurements showed statistically significant weak positive correlation in our study. As a result, the differences between minimum and maximum SVC measurements were relatively constant across different CVP values, thus producing no relationship between the collapsibility index and CVP.

Our study results also indicated a statistically significant weak positive correlation between minimum and maximum SVC diameters and thermodilution cardiac index, and a nonsignificant correlation between SVC collapsibility index and thermodilution cardiac index. This is an expected finding in liver transplant patients with a pathophysiology notable for low systemic vascular resistance and high cardiac output. Previous studies attempting to correlate several uncalibrated and calibrated arterial pressure-based cardiac output monitor-based measurements with thermodilution-obtained cardiac index showed lack of correlation.^23^, ^24^, ^25^ Furthermore, TEE measurements and thermodilution based cardiac index measurements were also found not to be interchangeable.^26^

The SVC collapsibility index was shown to be an excellent indicator of fluid responsiveness in mechanically ventilated septic patients in a previous study.^8^ The SVC collapsibility threshold to allow discrimination of fluid responsiveness was 36%. In the current study, the median [Q1, Q3] SVC collapsibility index was 25.5 [17.6, 33.0], indicating that most measurements were below the fluid responsiveness threshold. This can be explained by the active ongoing fluid resuscitation that occurs during liver transplant surgery in an effort to maintain euvolemia.

In recent years, SVC collapsibility index measurements using TEE to predict fluid responsiveness have been studied in major vascular surgery patients^8^ and in gastrointestinal surgery patients.^27^ The former study used miniature monoplane probes to allow continuous intraoperative measurement of fluid responsiveness, while the latter study, similar to our study approach, utilized omniplane probes for intermittent measurements. The major disadvantage of using monoplane probes is their inability to provide the full cardiac assessment that is required for the safe perioperative management of patients undergoing major vascular procedures, including obtaining essential transesophageal views for assessment of biventricular function and the presence of regional wall motion abnormalities.

### Limitations

Our study strengths include the novel concept of using noninvasive TEE derived SVC measurements and the uniform patient population studied. The study limitations include the lack of generalizability of our study results to other institutions since this is a single center study. Due to the advanced expertise in perioperative TEE required to perform intraoperative TEE measurements, reaching the needed sample size took several years and resulted in exclusion of patients from the study when the anesthesiology staff could not perform these measurements. This reduced the sample size and in turn reduced the power of the study. In addition, the TEE expertise required further limits the generalizability of the study results.

## Conclusions

This study showed a statistically significant weak positive correlation between both minimum SVC measurements, maximum SVC measurements, and CVP. It also showed a statistically significant weak positive correlation between both minimum SVC measurements, maximum SVC measurements and thermodilution cardiac index measurements. While statistically significant, the weak clinical correlation of intraoperative SVC measurements obtained by TEE make them unsuitable as a replacement for CVP or thermodilution cardiac index measurements in liver transplant recipients. Further studies should focus on assessment of noninvasive or minimally invasive hemodynamic parameters that can be reliably and continuously measured perioperatively in the liver transplant population and their correlation with established and validated invasive hemodynamic measurements including thermodilution cardiac output and index measurements.

## Ethics

Ethical approval for this study (IRB#:16-307) was provided by the Institutional Review Board at Cleveland Clinic, Cleveland, OH (Executive director: Bridget Howard, Esq, CIP) on March 7^th^, 2016

Our study was registered at ClinicalTrials.gov (NCT02818218; principal investigator M.A.; date of registration June 29, 2016) before the enrollment of the first patient (https://clinicaltrials.gov/study/NCT02818218?titles=SVC% 20 collapsibility%20index&rank=1).

## Funding

No funding was received for conducting this study. The authors have no relevant financial or non-financial interests to disclose.

## Conflicts of interest

The authors declare no conflicts of interest.

## Appendix Supplementary materials

Image, application 1

## Footnotes

Supplementary material associated with this article can be found in the online version at doi:10.1016/j.bjane.2024.844563.
