# Supplementary material for: Assessment of superior vena cava diameter and collapsibility index in liver transplantation: a prospective observational study
Source: Braz J Anesthesiol. 2024 Oct 9;74(6):844563. doi: 10.1016/j.bjane.2024.844563 (PMC11693420; doi:10.1016/j.bjane.2024.844563)
Supplement: Supplementary file 1 [file mmc1.docx]

BJAN-D-24-00384_Supplementary Material

**Supplemental Table 1** Complete cases analysis.

| **Measurement 1** | **Measurement 2** | **r_rm_ (98.3% CI)** | **p** | **n** |
| --- | --- | --- | --- | --- |
| Central Venous Pressure (mmHg) | SVC Collapsibility Index | -0.02 (-0.21, 0.16) | 0.754 | 210 |
|  | Minimum SVC (mm) | 0.27 (0.09, 0.43) | <0.001 |  |
|  | Maximum SVC (mm) | 0.33 (0.15, 0.48) | <0.001 |  |

The primary analysis was re-computed using only the 42 patients with complete SVC and CVP data.

**Supplemental Table 2** Bootstrapped estimates.

| **Measurement 1** | **Measurement 2** | **r (98.3% CI)** | **p** | **n** |
| --- | --- | --- | --- | --- |
| Central Venous Pressure (mmHg) | SVC Collapsibility Index | -0.06 (-0.22, 0.10) | 0.45 | 268 |
|  | Minimum SVC (mm) | 0.26 (0.05, 0.44) | 0.01 |  |
|  | Maximum SVC (mm) | 0.29 (0.11, 0.46) | <0.001 |  |

Confidence intervals for the Pearson’s correlation coefficients were constructed by 10,000 bootstrap samples. Point estimates are the overall sample correlations.

**Supplemental Table 3** STROBE Statement – Checklist of items that should be included in reports of *cohort studies.*

|  | Item No | Recommendation | Page No |
| --- | --- | --- | --- |
| Title and abstract | 1 | (a) Indicate the study’s design with a commonly used term in the title or the abstract | 1 |
|  |  | (b) Provide in the abstract an informative and balanced summary of what was done and what was found | 1, 2 |
| Introduction | | | |
| Background/rationale | 2 | Explain the scientific background and rationale for the investigation being reported | 3 |
| Objectives | 3 | State specific objectives, including any prespecified hypotheses | 4 |
| Methods | | | |
| Study design | 4 | Present key elements of study design early in the paper | 5 |
| Setting | 5 | Describe the setting, locations, and relevant dates, including periods of recruitment, exposure, follow-up, and data collection | 5 |
| Participants | 6 | (a) Give the eligibility criteria, and the sources and methods of selection of participants. Describe methods of follow-up | 5 |
|  |  | (b) For matched studies, give matching criteria and number of exposed and unexposed |  |
| Variables | 7 | Clearly define all outcomes, exposures, predictors, potential confounders, and effect modifiers. Give diagnostic criteria, if applicable | 6,7 |
| Data sources/ measurement | 8* | For each variable of interest, give sources of data and details of methods of assessment (measurement). Describe comparability of assessment methods if there is more than one group | 8,9 |
| Bias | 9 | Describe any efforts to address potential sources of bias | 9 |
| Study size | 10 | Explain how the study size was arrived at | 9 |
| Quantitative variables | 11 | Explain how quantitative variables were handled in the analyses. If applicable, describe which groupings were chosen and why | 10, 11 |
| Statistical methods | 12 | (a) Describe all statistical methods, including those used to control for confounding | 10, 11 |
|  |  | (b) Describe any methods used to examine subgroups and interactions |  |
|  |  | (c) Explain how missing data were addressed |  |
|  |  | (d) If applicable, explain how loss to follow-up was addressed |  |
|  |  | (e) Describe any sensitivity analyses |  |
| Results | | |  |
| Participants | 13* | (a) Report numbers of individuals at each stage of study ‒ e.g., numbers potentially eligible, examined for eligibility, confirmed eligible, included in the study, completing follow-up, and analyzed | 10 |
|  |  | (b) Give reasons for non-participation at each stage |  |
|  |  | (c) Consider use of a flow diagram | Figure 1 |
| Descriptive data | 14* | (a) Give characteristics of study participants (e.g., demographic, clinical, social) and information on exposures and potential confounders | Page 10, 11 (Table 1‒4) |
|  |  | (b) Indicate number of participants with missing data for each variable of interest |  |
|  |  | (c) Summarize follow-up time (eg, average and total amount) |  |
| Outcome data | 15* | Report numbers of outcome events or summary measures over time | Page 10, 11 |
| Main results | 16 | (a) Give unadjusted estimates and, if applicable, confounder-adjusted estimates and their precision (e.g., 95% Confidence Interval). Make clear which confounders were adjusted for and why they were included |  |
|  |  | (b) Report category boundaries when continuous variables were categorized |  |
|  |  | (c) If relevant, consider translating estimates of relative risk into absolute risk for a meaningful time period |  |
| Other analyses | 17 | Report other analyses done – e.g., analyses of subgroups and interactions, and sensitivity analyses | Page 11 (Table 5) |
| Discussion | | | |
| Key results | 18 | Summarize key results with reference to study objectives | 12 |
| Limitations | 19 | Discuss limitations of the study, taking into account sources of potential bias or imprecision. Discuss both direction and magnitude of any potential bias | 15 |
| Interpretation | 20 | Give a cautious overall interpretation of results considering objectives, limitations, multiplicity of analyses, results from similar studies, and other relevant evidence | 12‒14 |
| Generalizability | 21 | Discuss the generalizability (external validity) of the study results | 15 |
| Other information | | | |
| Funding | 22 | Give the source of funding and the role of the funders for the present study and, if applicable, for the original study on which the present article is based | Internal funding |
